# Neural Derivates of Canine Induced Pluripotent Stem Cells-Like Cells From a Mild Cognitive Impairment Dog

**DOI:** 10.3389/fvets.2021.725386

**Published:** 2021-11-04

**Authors:** Abinaya Chandrasekaran, Barbara Blicher Thomsen, Jørgen Steen Agerholm, Laís Vicari de Figueiredo Pessôa, Naira Caroline Godoy Pieri, Vahideh Sabaghidarmiyan, Katarina Langley, Miriam Kolko, André Furugen Cesar de Andrade, Fabiana Fernandes Bressan, Poul Hyttel, Mette Berendt, Kristine Freude

**Affiliations:** ^1^Department of Veterinary and Animal Sciences, Faculty of Health and Medical Sciences, University of Copenhagen, Frederiksberg, Denmark; ^2^Department of Veterinary Clinical Sciences, Faculty of Health and Medical Sciences, University of Copenhagen, Frederiksberg, Denmark; ^3^Department of Veterinary Medicine, Faculty of Animal Science and Food Engineering, University of São Paulo, Pirassununga, Brazil; ^4^Department of Drug Design and Pharmacology, University of Copenhagen, Copenhagen, Denmark; ^5^Department of Animal Reproduction, School of Veterinary Medicine and Animal Science, University of São Paulo, Pirassununga, Brazil

**Keywords:** dog, iPSCs, cognitive dysfunction, neurons, ciPSCLC

## Abstract

Domestic dogs are superior models for translational medicine due to greater anatomical and physiological similarities with humans than rodents, including hereditary diseases with human equivalents. Particularly with respect to neurodegenerative medicine, dogs can serve as a natural, more relevant model of human disease compared to transgenic rodents. Herein we report attempts to develop a canine-derived *in vitro* model for neurodegenerative diseases through the generation of induced pluripotent stem cells from a 14-year, 9-month-old female West Highland white terrier with mild cognitive impairment (MCI). Canine induced pluripotent stem cells-like cells (ciPSCLC) were generated using human OSKM and characterized by positive expression of pluripotency markers. Due to inefficient viral vector silencing we refer to them as ciPSCLCs. Subsequently, the ciPSCLC were subjected to neural induction according to two protocols both yielding canine neural progenitor cells (cNPCs), which expressed typical NPC markers. The cNPCs were cultured in neuron differentiation media for 3 weeks, resulting in the derivation of morphologically impaired neurons as compared to iPSC-derived human counterparts generated in parallel. The apparent differences encountered in this study regarding the neural differentiation potential of ciPSCLC reveals challenges and new perspectives to consider before using the canine model in translational neurological studies.

## Introduction

Neurodegenerative diseases represent a substantial unmet medical need, notably as the population ages worldwide. Many neurodegenerative diseases, including Alzheimer's disease (AD) and Parkinson's disease lack any significant or truly effective treatments. Precisely understanding the pathological mechanisms of these diseases is a key step for developing disease-modifying drugs able to prevent or at least slow their progression. AD is an irreversible, currently untreatable, neurodegenerative disorder affecting ~50 million people worldwide (1, 2). AD is characterized by two distinct hallmarks: Accumulation of extracellular amyloid plagues containing Amyloid beta (Aβ) peptides and intracellular neurofibrillary tangles (NFTs) containing hyperphosphorylated forms of TAU (3). Unfortunately, so far, all attempts to develop drugs to intervene into neuronal degeneration have failed in stage one and two clinical trials. The majority of these potential drugs have been tested for their efficacy using transgenic mouse models, which artificially express human genes containing mutations identified in early onset familiar AD (fAD). These genes include Presenilin 1 (PSEN1), Presenilin 2 (PSEN2), and Amyloid Precursor Protein (APP) (4). Depending on the different transgenic models the hallmarks of AD can be more or less faithfully recapitulated. Some models recapitulate altered APP processing (5–7), whilst they do not develop amyloid plagues and NFTs (8). Other very aggressive models, such as the 5xfAD mice harboring 5 human transgenes (3 copies of human APP with the Swedish (K670N, M671L), Florida (I716V), and London (V717I) mutations and two copies of human PSEN1 with M146L and L286V mutations), display many pathological AD hallmarks, such as amyloid plagues, synaptic degeneration, and microglia activation. However, none of these models, not even the most aggressive 5xfAD model, develops NFTs (9). One potential reason for the lack of NFT formation could be attributed to the short lifespan of rodents, which may not allow a long pre-symptomatic disease phase as seen in man. Even though several studies have reported the presence of NFTs using transgenic mouse models (10–12), it should be noted that such analyses were based on accumulated inter-neuronal hyperphosphorylated TAU detected by immunocytochemistry (ICC), which can only indicate the presence of pre-tangles. Genuine NFTs presence is defined as neurofibrillary pathology with tangles in the in cell bodies of neurons [reviewed in (13)]. Moreover, amyloid plagues in rodent AD models appear more diffuse with less cross-linked fibrils (14), which again could be caused by the relative short time span of disease progression. Hence, species differences exist with regards to formation of amyloid plagues and, in particular, NFTs, and these reports indicate that mouse models of AD are of suboptimal translational value, even though it is important to acknowledge their important role in identifying mechanistic insights of fAD mutations. Consequently, it would be more appropriate to study neurodegenerative diseases as e.g., AD in animal model species, which develop age depended cognitive decline comparable to AD in man (natural models). Such species would have longer life-spans and be exposed to similar environmental conditions as their human counterparts. Amongst such natural models, dogs are unique since a certain proportion of aged dogs spontaneously develop dementia, a condition termed canine cognitive dysfunction (CCD) (15). Studies on this condition are still relatively sparse and sometimes contradicting, but affected dogs have been shown to exhibit some forms of AD-like neuropathology (16). In addition to the accumulation of Aβ and, rarely NFTs (17), cortical atrophy, neuronal loss, reduced neurogenesis, and cerebral amyloid angiopathy have been reported (18–20). Clinically, dogs with CCD exhibit signs comparable to AD patients, including slowly progressing changes in social interactions, signs of disorientation, impaired memory and learning as well as changes in the level of activity (21).

CCD has been reported to affect ~20% of dogs older than 8 years and more than half of dogs older than 15 years (21). These dogs exhibit a clinical phenotype with similarities to human AD including slowly progressing changes in social interactions, signs of disorientation, impaired memory, and learning as well as changes in the level of activity (21). A milder (prodromal stage) of CCD, mild cognitive impairment (MCI), also occurs in older dogs and is characterized by some of the same changes in behavior and daily routines as registered with CCD but in a less pronounced form. MCI in dogs is not directly comparable to human MCI, but may, however, appear as a prodromal phase of CCD. The prevalence of MCI in dogs is unknown but it is considered to be a common condition in aging dogs (22). One study found that over the course of 24 months, 22% of dogs with MCI progressed to having CCD (23). MCI in dogs may correspond to MCI in humans, where 22.2% of people older than 71 years have been estimated to be affected (17, 22). Humans diagnosed with MCI have a decline in cognitive abilities, but preserve their ability to maintain a normal life. The diagnosis of MCI poses individuals at an increased risk of developing AD or other types of dementia (15). For these reasons, we propose to further develop the companion dog with CCD as a model for neurodegenerative disease. In this study, fibroblasts from a 14-year-old dog suffering from MCI were reprogrammed into canine induced pluripotent stem cells-like cells (ciPSCLC), followed by induction into neural precursor cells (NPCs) and early stage neurons. This *in vitro* model of neuronal commitment could be an attractive model to study early brain-cell type specific disease pathology in the canine model with the potential to extrapolate the findings to neurodegenerative disease in man.

## Materials and Methods

### Study Design

#### Dog Recruitment and Assessment

A skin biopsy from a client-owned dog affected by MCI was used for the study. The dog was recruited from a cohort of older dogs participating in an observational study of cognitive dysfunction at the University Hospital for Companion Animals, Department of Veterinary Clinical Sciences, University of Copenhagen.

The handling of animals was performed according to the EU directive on handling and protection of animals used for scientific purposes (2010/63/EU) and the study was approved by the Ethical Committee of the Department of Veterinary Clinical Sciences, University of Copenhagen, Denmark (Permission number 2015-15-0201-00810 & 2017-7). Written consent was obtained from the owners.

#### *In vitro* Experiment Design

The cellular work was designed and conducted at the Department of Veterinary and Animal Sciences, University of Copenhagen.

### Patient Description

A 14-year, 9-month-old female West Highland White Terrier was referred to the University Hospital due to a 6 months history of behavioral changes. The owner reported disorientation and changes in the dog's social interaction (less interested in greeting other dogs, less interested in physical contact and following the owner closely). The dog furthermore showed signs of separation anxiety, changes in sleep-wake cycles (waking 4–5 times every night and pacing before lying down again), and changes in the level of activity (reluctant to go for walks and no longer wanting to explore things in the garden). As a surprise to the owner, the dog had since the age of 10 years suddenly started to play with toys again. The dog was reported to be otherwise well with no previous medical problems except for a suspected lower back problem not further evaluated.

### Clinical and Paraclinical Assessment

The dog was examined according to a standard protocol, including a clinical and neurologic examination, hematology including complete blood count, serum biochemistry, C-reactive protein, thyroid profiles including TSH, total T4, and free T4 as well as a full urinalysis. Blood analyses were performed at the Central Laboratory, Department of Veterinary Clinical and Animal Sciences, University of Copenhagen. Here, no abnormalities where detected which could explain the behavioral changes observed in the dog.

### Questionnaire Investigation

CCD as well as MCI are clinical diagnoses established by prior exclusion of differential diagnoses such as brain tumors. When establishing a diagnosis, the clinical and neurological examination as well as appropriate paraclinical tests used in combination with CCD-specific questionnaires, such as the Canine Cognitive Dysfunction Rating scale (CCDR) (24), are crucial (21). The CCDR questionnaire includes questions regarding 13 specific behavioral changes. Each question contains five different response options with a corresponding score ranging from 1 to 5 that reflects the severity of the observed behavioral change. When using the CCDR questionnaire, non-demented dogs will have a score equivalent of 0–39, dogs with MCI will have a score of 40–49, and dogs with CCD will have a score of 50–80 (24, 25).

#### Dementia Score

The dogs cognitive status was assessed using the CCDR questionnaire (24). The dog had a CCDR score of 42, equivalent to mild cognitive impairment.

#### Follow-Up

One year later the dog developed pronounced separation anxiety, unwilling to go for walks, increased disorientation at home, easily getting scared. The CCDR score had at this time increased to 48 (which is very close to fulminant CCD). Euthanasia was performed for welfare reasons following the hospital guidelines at the age of 14 years and 9 months. A skin biopsy obtained immediately upon death was used for this study to generate iPSC as described below.

#### Necropsy Findings

The dog was necropsied shortly after euthanasia. The brain was submerged *in toto* in 10% neutral buffered formalin, fixed for 3 weeks, embedded in agar and sliced in 6 mm parallel coronal sections. Except for leptomeningeal fibrosis in the cerebral sulci, no other gross lesions were observed in the brain. Disseminated greenish-brown foci, which histologically consisted of infiltrating hemosiderin-laden macrophages and loss of parenchyma, were present in the liver. In the lung, multifocal inert calcifications, and granulomatous bronchio-alveolitis were seen. The liver and lung findings indicated previous visceral larvae migrans.

### Clinical Assessment

#### Initial Physical and Neurological Examinations

At time of presentation, the dog was bright and alert. The dog lost its balance on the hind legs a few times, primarily the right hind, and had a sore lower back and psoas musculature. No other abnormalities were detected on physical and neurological examinations, and the loss of balance was suspected to be caused by an underlying condition in the spinal cord. The owner declined further diagnostic investigations as the dog was only mildly affected.

#### Paraclinical Investigations

No abnormalities were detected on hematology, biochemistry including bile acids, thyroid profile, and full urinalysis.

### Reagents

The cell culture reagents and culture plates were purchased from Stem Cell Technologies and Thermo Fisher Scientific unless specified otherwise.

### Fibroblast Isolation

Fibroblasts were isolated from a skin biopsy obtained from inguinal region via surgical procedures, previously shaved and disinfected. The skin biopsy was rinsed in sterile PBS solution containing 2% penicillin and streptomycin (Gibco, 15070-063). Biopsy was finely minced with sterile scalpel blades and digested in 5 mL collagenase IV 0.1% (Sigma, C2674) for 3 h, at 38.5°C. After digestion, the sample pellet was rinsed with DMEM (Sigma, D8437) with 10% FBS (Gibco, 10091148), and 1% penicillin and streptomycin and cells were subsequently plated on gelatin coated plastic ware. Cells were cultured at 95% humidity, at 38.5°C, and 5% CO_2_. Media was replaced every other day, and cells were dissociated and re-plated (TrypLE Select Gibco, A12177-01) before reaching 90% confluence, expanded until passage 3 and cryopreserved for further studies.

### Cellular Reprogramming

Canine fibroblasts from one healthy 4 year dog, one MCI 14 year old dog and human fibroblasts from A79V mutation with PSEN1 were subjected to *in vitro* reprogramming.

Human induced pluripotent stem cells (hiPSCs) were generated via episomal reprogramming from human dermal fibroblasts obtained from skin biopsy. Skin fibroblasts were obtained from a 48-year-old presymptomatic woman carrying an A79V mutation in the presenilin 1 gene (PSEN1), causing Alzheimer's disease (AD). Induced pluripotent stem cell (iPSCs) were derived via transfection with episomal vectors carrying hOCT4, hSOX2, hKLF2, hL-MYC, hLIN28, and shTP53 genes. 1 × 10 ^5^ fibroblasts were electroporated with a total of 1 μg plasmids carrying the sequences for hOCT4, hSOX2, hKLF4, hL-MYC, and hLIN28 with or without a short hairpin against TP53 (shp53) and cultured in fibroblast medium. Seven days after electroporation, the fibroblasts were trypsinized and split 1:2 onto hESC-qualified Matrigel-coated dishes (BD Biosciences, NJ, USA) and cultured in E8 medium (Life Technologies) in 5% O2, 5% CO2 in N2 with the medium replenished every other day. Emerging iPSC-like colonies were manually picked and further characterized. The cell line A79V mutation has been previously described (26). In addition to the A79V mutation, we have included the gene corrected line, A79V-GC-hiPSCs line to our study. The generated A79V mutation-hiPSC (c.236 C>T) was gene corrected with CRISPR-Cas9 gene editing by substituting the point mutation “T” with the wild-type nucleotide “C.” This gene-corrected line could potentially, serve as an isogenic control to the mutant line for future investigation of mechanisms and cellular phenotypes altered by this specific PSEN1 mutation. The cell line A79V-GC has also been previously described (27).

Canine fibroblasts from a healthy control dog (4 years-old) were submitted to episomal reprogramming, in duplicate, following the methodology described above for human cell reprogramming. In brief, cells were maintained under fibroblast conditions until day 6 (D6) post-electroporation, when they were reseeded and divided into two groups: in first group (G1), the cells were maintained in Matrigel-coated dishes (BD Biosciences, NJ, USA) and cultured in E8 medium (Life Technologies) and in the second group (G2) they were cultured onto MEFs (inactivated murine embryonic fibroblasts, Millipore) “iPSCs media,” composed of Knockout Dulbecco's modified Eagle's medium F12 (DMEM) (Gibco), 20% Knockout Serum Replacement (KSR - Gibco,10828-028), 1% penicillin and streptomycin (Gibco, 15070-063), 1% Glutamax (Gibco, 35050-0610), 1% non-essential amino acids (NEAAs; Gibco, M7145) and 3.85 μM b-mercaptoethanol (Gibco, 31350-010) and supplemented with 10 ng/mL human bFGF (PEPROTECH, 100-18B). Medium was refreshed every other day and cells were maintained in culture for ~3 weeks.

The lentiviral reprogramming protocol for ciPSCs generation was performed as previously described for dogs and other species with minor modifications (28–30). Briefly, 90% confluent 293FT cells (Thermo Fisher, R70007) were transduced with human OSKM (STEMCCA, Millipore/Sigma, SCR545), VSVG and remaining auxiliary vectors (TAT, REV, hgpm2; proportion of 6:2:1:1:1, respectively) through lipofection (Thermo Fisher, L3000001) and incubated. Culture media containing viral particles was collected after 48 and 72 h, filtrated, and used for transductions of canine cells, in the presence of 8 μg/mL hexadimethrine bromide (Polybrene - Sigma, H9268).

For cellular reprogramming, 3 × 10^4^ canine fibroblasts were plated 24 h prior to the first transduction and cultured in DMEM, as described above. After 24 and 48 h of initial plating, canine fibroblasts were transduced overnight with 1 mL of filtrated viral particles and polybrene. Six days after first transduction, 2 × 10^4^/9.5 cm^2^ were re-plated onto 6 wells dishes coated with a feeder layer of 2 × 10^5^ mitomycin C (Sigma, M4287) treated mouse embryonic fibroblasts (MEFs) and cultivated in “iPSCs media” supplemented with 10 ng/mL LIF (PROSPEC, CYT-644-b) and replaced every other day. Emerging iPSC-like colonies were manually picked and labeled as passage 1; and clonal lines were expanded using UltraPure™ 0.5 mM EDTA (Invitrogen, 15575-020) for further characterization and cryopreservation.

### Alkaline Phosphatase (AP) Activity Detection

ciPSCLC were washed three times with PBS and fixed in 4% paraformaldehyde for 5–7 min at room temperature (RT). AP activity was then assayed with NBT/BCIP chromogen solution (Roche, Basel, Switzerland).

### Canine Neural Induction

Canine neural progenitor cells (cNPCs) were generated from the ciPSCLC either by (i) inducing the cells with 20 ng/mL epidermal growth factor (EGF) and 20 ng/mL basic fibroblast growth factor (bFGF) selection method for 10 days (using Neural Induction Medium I, detailed in Table 1A) or with (ii) 50 ng/mL Noggin (R&D) and 20 ng/mL EGF selection method for 10 days (using Neural Induction Medium II, detailed in Table 1B). Both protocols were tested according to a previous publication (31). Neural rosettes were manually picked and re-plated onto Matrigel plates. At passage 4, the cells were seeded as single cells (20.000 cells/cm^2^) using Accutase and NPCs were further cultured and expanded in neural maintenance medium (NMM), detailed in Table 1C. For terminal differentiation the cNPCs were plated on poly-ornithine laminin plate with neural differentiation medium (NDM), detailed in Table 1D for 3 weeks.

**Table 1:** **(A)** Neural Induction Medium, **(B)** Neural Induction Medium II, **(C)** Neural Maintenance Media (NMM), **(D)** Neura Differentiation Medium (NDM).

**Reagents**	**Company, catalog number**	**Final concentration V = 50 ml**
**(A)**		
Neurobasal medium, L-Glutamine (100X)	Gibco, 2161553	50 ml
B27, minus vitamin A (RA) (100X)	Life Technologies, 12587-010	1 ml
N-2 (100X)	Gibco, 17502-048	500 μl
Non-essential amino acid (100X)	Sigma-Aldrich, M7145	500 μl
GlutaMAX Supplement (100X)	Gibco, 35050038	500 μl
Penicillin-Streptomycin (100X)	Sigma-Aldrich, P0781	500 μl
Noggin (500ng/mL)	R&D, 6057-NG	50 ng/μl
Epidermal Growth Factor (EGF) (10 μg/mL)	Prospec, Cyt-217	20 ng/μl
**(B)**		
Neurobasal medium, L-Glutamine (100X)	Gibco, 2161553	50 ml
B27, minus vitamin A (RA) (100X)	Life Technologies, 12587-010	1 ml
N-2 (100X)	Gibco, 17502-048	500 μl
Non-essential amino acid (100X)	Sigma-Aldrich, M7145	500 μl
GlutaMAX Supplement (100X)	Gibco, 35050038	500 μl
Penicillin-Streptomycin (100X)	Sigma-Aldrich, P0781	500 μl
Fibroblast Growth Factor (bFGF) (20 μg/mL)	PeproTech, 100-18b	20 ng/μl
Epidermal Growth Factor (EGF) (100 μg/mL)	Prospec, Cyt-217	20 ng/μl
**(C)**		
Neurobasal medium, L-Glutamine (100X)	Gibco, 2161553	50 ml
B27, minus vitamin A (RA) (100X)	Life Technologies, 12587-010	1 ml
N-2 (100X)	Gibco, 17502-048	500 μl
Non-essential amino acid (100X)	Sigma-Aldrich, M7145	500 μl
GlutaMAX Supplement (100X)	Gibco, 35050038	500 μl
Penicillin-Streptomycin (100X)	Sigma-Aldrich, P0781	500 μl
Fibroblast Growth Factor (bFGF) (20 μg/mL)	PeproTech, 100-18b	10 ng/μl
Epidermal Growth Factor (EGF) (100 μg/mL)	Prospec, Cyt-217	10 ng/μl
**(D)**		
Neurobasal medium, L-Glutamine (100X)	Gibco, 2161553	50 ml
B27, minus vitamin A (RA) (100X)	Life Technologies, 12587-010	1 ml
N-2 (100X)	Gibco, 17502-048	500 μl
Non-essential amino acid (100X)	Sigma-Aldrich, M7145	500 μl
GlutaMAX Supplement (100X)	Gibco, 35050038	500 μl
Penicillin-Streptomycin (100X)	Sigma-Aldrich, P0781	500 μl
Ascorbic acid (20 mM)	Sigma-Aldrich, A4403	500 μl
Dibutyryl-cAMP (50 mM)	Sigma-Aldrich, D0627	50 μl
Brain-derived neurotrophic factor (BDNF)	Prospec, Cyt-207	20 ng/μl
Glial-derived neurotrophic factor (GDNF)	Prospec, Cyt-305	20 ng/μl

### Human Neural Induction

Human neural progenitor cells (hNPCs) were generated from the human iPSCs (hiPSC) by dual inhibition of SMAD signaling pathway using LDN193189, an inhibitor of the BMP pathway (Selleck, S2618) and SB431542, a small molecule inhibitor of the TGFß pathway (Selleck, S1067) in accordance to our previously published protocol (32). The hNPCs were expanded and analyzed in a similar way as the cNPCs but with human neural maintenance medium (NMM), detailed in (32). For terminal differentiation the hNPCs were plated on poly-ornithine laminin plate with human neural differentiation medium (NDM) as previously published in (32).

### Immunocytochemistry and Confocal Microscopy

Canine and human cells (i.e., iPSCs, NPCs, and neurons) were fixed in 4% paraformaldehyde (PFA) (20 min, RT), rinsed 3 times in PBS and permeabilized (0.2% triton X-100 in PBS; 20 min). After blocking for 30 min (at RT in 5% donkey serum) the cells were incubated with primary antibodies (refer details and dilutions in Table 2) overnight at 4°C. On the following day, the isotype specific secondary antibodies (for details refer Table 2) were applied (1 h at RT). Samples were washed in DPBS and stained with DAPI (Sigma-Aldrich, D9542) to label the nuclei of the cells. Samples were visualized on a confocal microscope equipped with a Leica TCS SPE microsystem controlled by LAS X software (v 2.0.0.14332). Detailed information on antibodies is presented in Table 2.

**Table 2:** Antibodies used for immunocytochemistry.

	**Antibody**	Antibody Registry^*^ Identifier	**Dilution**	**Company**
Pluripotency	Mouse anti-SOX2	AB_2251134	1:1,000	Millipore
	Rat anti-OCT4	AB_2167713	1:100	R&D
	Goat anti-NANOG	AB_1268274	1:1,000	R&D
Neural differentiation	Mouse anti-NESTIN	AB_11211837	1:1,000	Millipore
	Rabbit anti-PAX6	AB_2313780	1:200	Biolegend
	Goat anti-SOX1	AB_2239879	1:50	R&D
	Mouse anti-ß-III-TUBULIN	AB_1119489	1:1,000	Sigma-Aldrich
	Rabbit anti-GFAP	AB_10013382	1:1,000	Dako
	Rabbit anti-APP	AB_445173	1:500	Abcam
	Rabbit anti-TAU	AB_10013724	1:500	Abcam
Secondary antibodies	AF 488 donkey anti-goat IgG	AB_2534102	1:2,000	TFS
	AF 488 donkey anti-mouse IgG	AB_2535788	1:2,000	TFS
	AF 488 donkey anti-rabbit IgG	AB_2535792	1:2,000	TFS
	AF 594 donkey anti-rabbit IgG	AB_2556547	1:2,000	TFS
	AF 594 donkey anti-goat IgG	AB_2534105	1:2,000	TFS
	AF 594 donkey anti-mouse IgG	AB_253578	1:2,000	TFS

*AF, Alexa Fluor; TFS, Thermo Fisher Scientific Inc*;

* *http://antibodyregistry.org/*.

### Nuclear Area Quantification

The ratio of nuclear to cytoplasmic (N/C) localization was quantified using the ImageJ intensity ratio nuclei cytoplasm tool (33). The threshold, select area, and ROI manager functions of ImageJ were used to reduce background as described previously (34). On an average, we measured the ratio of N/C localization in six to 10 fields, from three independent replicates, in both canine and human neural differentiations. The total number of cells was represented by the number of DAPI-labeled nuclei on each image. Data is represented in Mean ± SEM.

### Quantitative PCR

RNA was extracted using RNeasy^®^ Plus Mini Kit (Qiagen, 74134) according to the manufacturer's protocol. cDNA was synthesized from 1 μg of total RNA in 20 μL reaction using iScript™ cDNA synthesis Kit (BIO-RAD, 1708890). After synthesis, the cDNA was diluted four times with double distilled water and stored at −20°C. Quantitative RT-PCR (qPCR) reactions were done in triplicates using the FastStart Lightcycler 480 SYBR Green I Master (Roche, 04707516001) on LightCycler^®^ 480 real-time PCR system (Roche, Switzerland). cDNA samples were subjected to PCR amplification with primers listed in Table 3.

**Table 3:** Primers used for RT-qPCR.

**Gene Name**	**Gene ID**	**Forward**	**Reverse**	**References**
*cOCT3/4*	481709	TCAAAACCGCCTCAAGTTGG	CAGGGTGGGCTTCGGGCAC	XM_038682836
*cNANOG*	100856473	GGTACCTGCTGAACCCTTCT	GCAGCGATTCCTCTTCACAG	XM_038437912
*cSOX2*	488092	CCGAGTGGAAACTTTTGTCGG	TAGCTGTCCATGCGCTGGTT	XM_038445642
*cTUBB3*	102154194	ACACGCACCGAGCATGAGG	CCGAGGCACATACTTATGAGAAGA	XM_038666722
*cB-ACTIN*	487218	TGTGTTATGTGGCCCTGGAC	GGATTCCATGCCCAGGAAGG	AF023846
*hSTEMCCA(hOSKM)*	–	AAGAGGACTTGTTGCGGAAA	GGCATTAAAGCAGCGTATCC	(35)
*hOCT3/4*	5460	CCCCAGGGCCCCATTTTGGTACC	ACCTCAGTTTGAATGCATGGGAGAGC	NM_203289
*hNANOG*	79923	AAAGAATCTTCACCTATGCC	GAAGGAAGAGGAGAGACAGT	NM_001297698.2
*hSOX2*	6657	TTCACATGTCCAGCACTACCAGA	TCACATGTGTGAGAGGGGCAGTGTGC	NM_003106.4
*hTUBB3*	10381	AACGAGGCCTCTTCTCACAA	GGCCTGAAGAGATGTCCAAA	NM_001197181.2
^*^hGAPDH	2597	CTCTCTGCTCCTCCTGTTCGAC	TGAGCGATGTGGCTCGGCT	NM_001256799.3

* *was used as reference gene; c, canine; h, human*.

### Statistics

For all experiments, data are presented as mean ± standard errors of the mean (SEM). Statistical analysis was made in GraphPad Prism 7.03 and determined using Student's *t*-test; by one-way ANOVA with a Tukey's post-test with Bonferroni *post-hoc* test for differences of mean between each group, as indicated. Statistical significance was labeled in figures as ^*^ (*p* < 0.05) and ^**^ (*p* < 0.01).

## Results

### Generation of ciPSCLC

The attempt of deriving ciPSCs with a non-integrative reprogramming system yielded clusters of canine cells 21 days after electroporation. These clusters were manually re-plated at the first passage onto Matrigel coated 6 well-plates and enzymatically dissociated for following passages. Each cell cluster in passage 1 was cultured for 1 week. Cell clusters were tested for alkaline phosphatase (AP) and OCT4 expression. None of the colonies were AP or OCT4 positive. Three colonies were maintained in culture and split with trypsin-EDTA onto 6-well-plates for two additional weeks, however the cells did neither proliferate nor formed colonies again (Supplementary Figure 1). Although morphological changes were observed, episomal reprograming of control canine fibroblasts was not achieved and therefore the lentiviral reprogramming method was used in subsequent reprogramming attempts.

**Figure 1:**
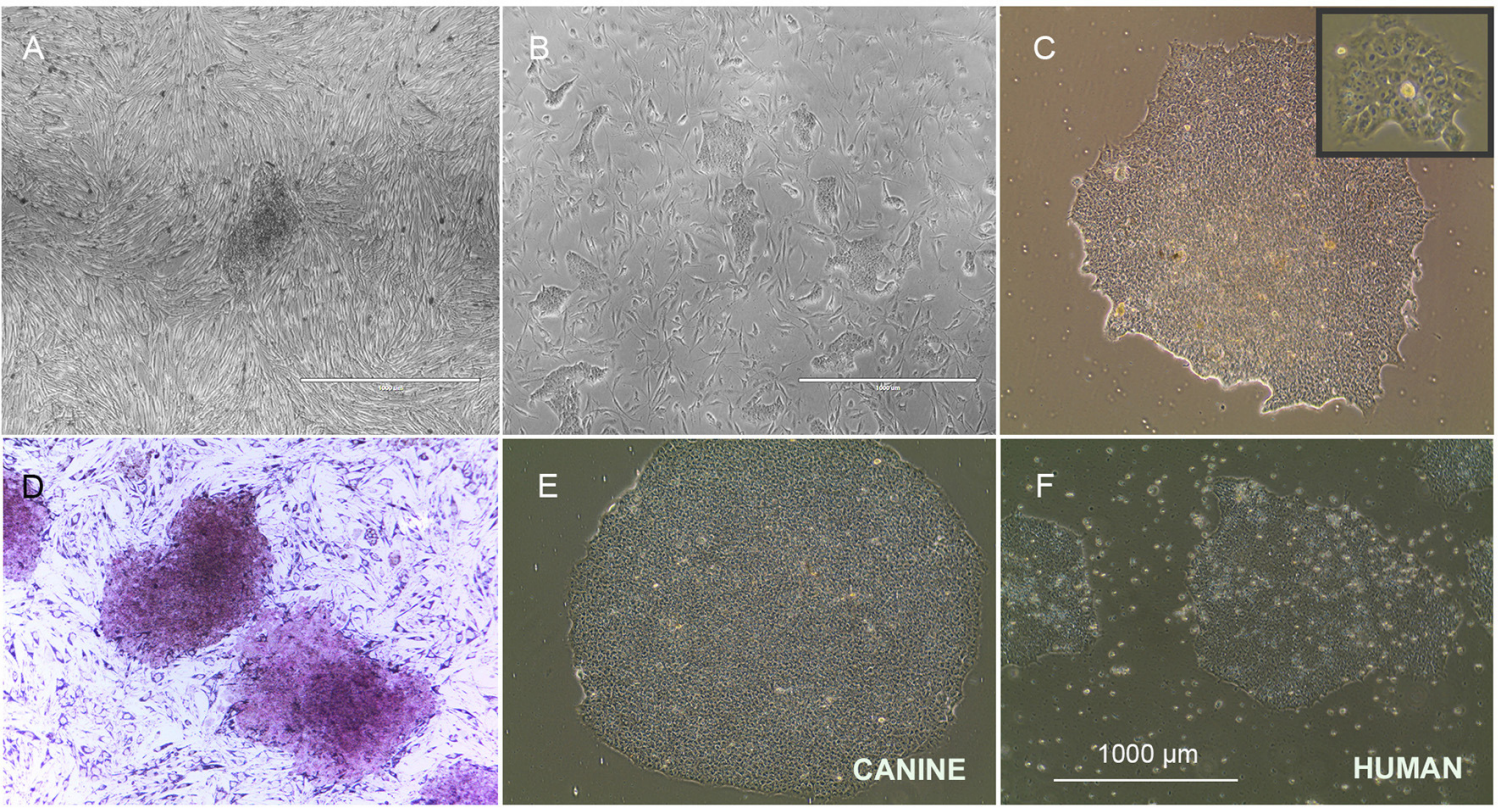
Morphology of ciPSCLC. **(A)** ciPSCLC colonies 12 days post transduction. **(B)** On day 28 post transduction (Passage 2), ciPSCLC colonies were moved onto mouse embryonic fibroblasts (MEF's) feeder layer. **(C)** On day 32 post transduction (Passage 3), ciPSCLC colonies were transferred onto Matrigel. Note the tighter packaging and the highly refractile cell morphology presenting high nuclear to cytoplasmic ratio. **(D)** Alkaline phosphatase (AP) staining of ciPSCLC. **(E,F)** Comparison of colonies of ciPSCLC vs. hiPSCs. Scale bar = 1,000 μm.

**Figure 2:**
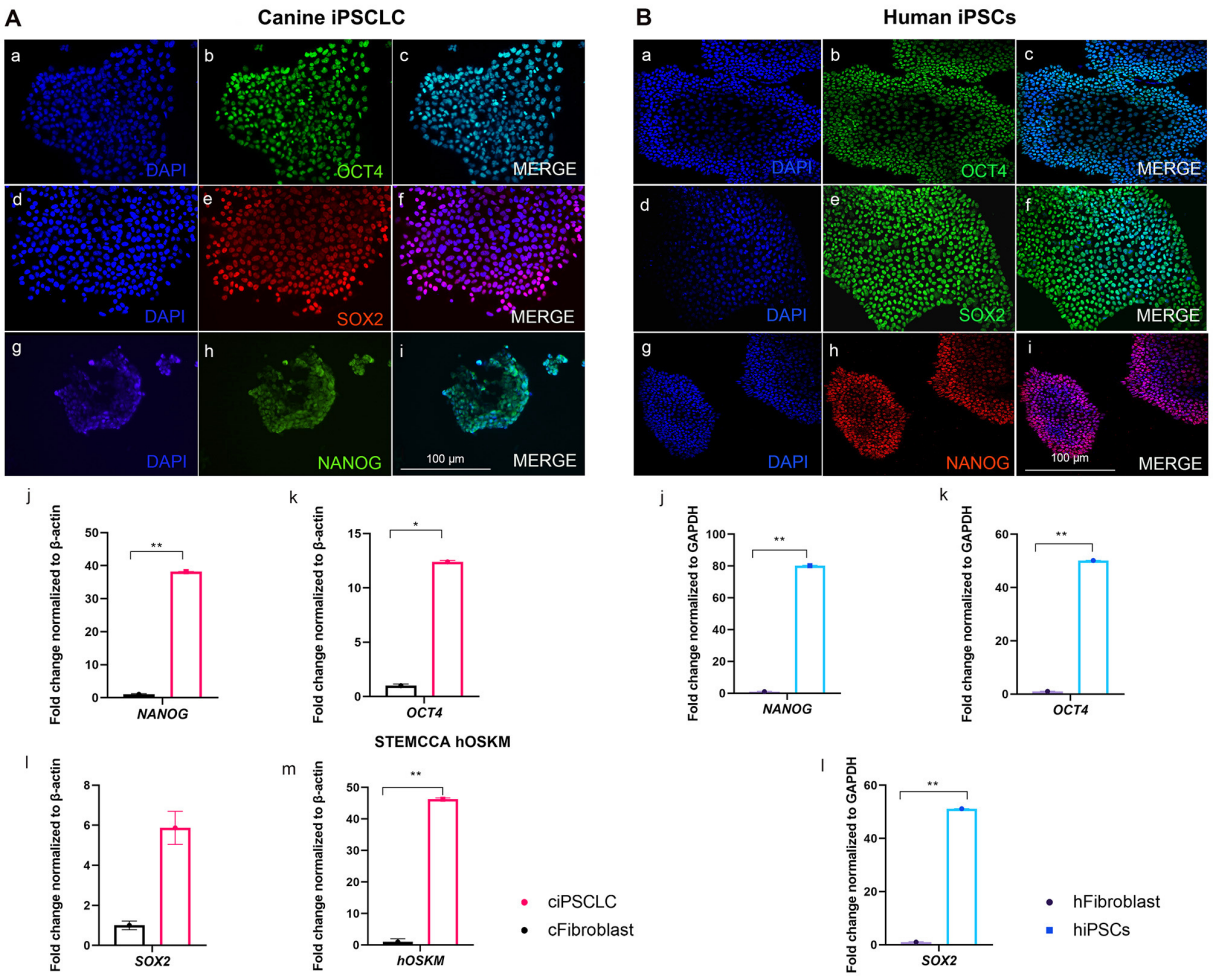
(**A**, left): Pluripotency marker characterization of ciPSCLC (Passage 10). (a–c) OCT4 (green). (d–f) SOX2 (red). (g–i) NANOG (green), DNA labeled with DAPI in blue. qPCR of NANOG (j), OCT4 (k), SOX2 (l), and STEMCCA [hOSKM; (m)]. (**B**, right): Pluripotency marker characterization of hiPSCs (Passage 10). (a–c) OCT4 (green). (d–f) SOX2 (green). (g–i) NANOG (red), DNA labeled with DAPI in blue. qPCR of NANOG (j), OCT4 (k), SOX2 (l). Scale bar =100 μm. ^*^ < 0.05; ^**^ < 0.001.

We have successfully reprogrammed adult canine dermal fibroblast into ciPSCLC using a lentiviral system (OSKM) expressing the human reprograming factors OCT4, SOX2, KLF-4, c-MYC. ciPSCLC were initially observed at day 12 post transduction and displayed characteristic hiPSCs-like morphology with a flat shape colony appearance and densely packed individual colonies with high nucleus-to-cytoplasm ratio (Figure 1A). Around 18 days after transduction, colonies grew large enough to be mechanically isolated and transferred onto fresh mitomycin C-inactivated embryonic fibroblast (MEFs) (Figure 1B). After on average three stable passages onto MEFs, colonies were manually selected and transferred onto Matrigel (Figure 1C). Sixteen sub-clones were generated from the original canine fibroblast line. From these 16 ciPSCLC lines, 3 lines were selected for pluripotency marker expression analyses. Alkaline phosphatase activity was detected in ciPSCLCcolonies from all 3 clones, indicating pluripotency (Figure 1D). The proliferative activity and morphology of the ciPSCLC line was similar to the previous published report (36); and the ciPSCLC were morphological identical to hiPSCs (Figures 1E vs. 1F). Next, we evaluated the differentiation capacity of ciPSCLC in embryoid bodies (EBs). EBs of ciPSCLC failed to display characteristic EB morphology over a 14-day period, suggesting a lack of differentiation capacity. The hiPSCs used in these experiments as a positive control for proper EB formation have previously been published and displayed full pluripotent characteristics (26, 27). To determine the pluripotency status of our ciPSCLC at the protein expression level, we performed immunocytochemistry (ICC). ciPSCLC were positive for OCT4, SOX2, and NANOG confirming their pluripotency status (Figure 2Aa–i). In order to further verify pluripotency in our ciPSCLC at the transcript level, expression of key pluripotency genes was determined via reverse transcriptase PCR (qPCR) (Table 3). Furthermore, since our ciPSCLC have the human pluripotency factors stably integrated into their genome during the lentiviral reprogramming, we designed canine-specific primers for *NANOG, OCT4*, and *SOX2* to discriminate between human and canine-specific gene expression. ciPSCLC expressed the endogenous canine pluripotency markers, *NANOG, OCT4* and *SOX2* (Figure 2Aj–l), whereas the fibroblasts from which the ciPSCLC were generated, did not. The gene expression profiles of ciPSCLC and hiPSCs were clearly similar, but in general the gene expression levels were lower in the ciPSCLC compared to hiPSC (Figure 2Bj–l). Most importantly, we also checked for the exogenous expression of hOSKM in our generated ciPSCLC. Our results showed that the exogenous vector expression was observed in our ciPSCLC at pluripotent state indicating that the viral construct was not silenced (Figure 2Am). The hiPSC, on the other hand, were generated by the use of non-integrative episomal vector reprogramming and retention of episomal plasmids was no longer detectable after passage 10 (37).

### Efficient Neural Induction of ciPSCLC

**Figure 3:**
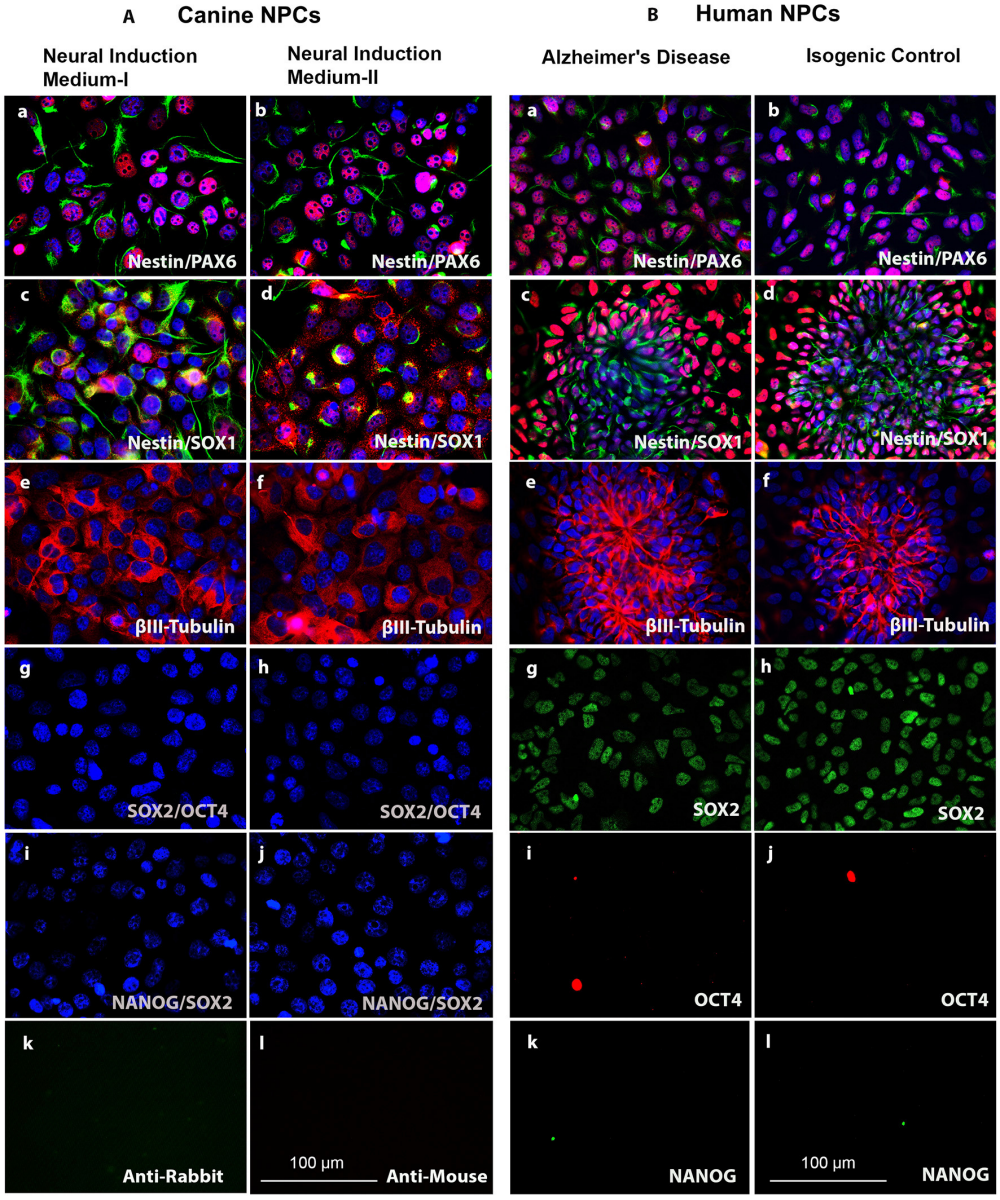
(**A**, left). Characterization of cNPCs between the Noggin/EGF (Neural Induction Medium I) and the bFGF/EGF (Neural Induction Medium II) neural induction methods as compared to human neural progenitor cells (hNPCs). (a,b) NPC markers Nestin (green) and PAX6 (red). (c,d) NPC markers Nestin (green) and SOX1 (red). (e,f) Early neural marker βIII-Tubulin (red). (g,h) Pluripotency markers SOX2 (green) and OCT4 (red). (i,j) Pluripotency markers NANOG (green) and SOX2 (red) and DNA labeled with DAPI in blue. (k,l) Negative control for ICC secondary antibody staining. (**B**, right) Characterization of human neural progenitor cells (hNPCs). (a,b), NPC markers Nestin (green) and PAX6 (red). (c,d) NPC markers Nestin (green) and SOX1 (red). (e,f) Early neural marker βIII-Tubulin (red). (g,h) Pluripotency marker SOX2 (green). (i,j) Pluripotency marker OCT4 (red). (k,l) Pluripotency marker NANOG (green) and DNA labeled with DAPI in blue. Scale bars = 100 μm.

In order to characterize the generated cNPCs, we investigated the expression of Nestin, PAX6, and SOX1. Nestin is an intermediate filament protein highly expressed in NPCs, PAX6 is transcription factor expressed in neuroectodermal development and an indicator of forebrain NPCs. SOX1 is a transcription factor belonging to the SOXB1 subgroup of the high mobility group box (HGM-box) family critically involved in the regulation of neural stem cell fate and pluripotent stem cell fate, respectively (38). NPCs were expanded for four passages, followed by ICC evaluation of the aforementioned NPC markers. Supporting evidence suggest that infusion of EGF and bFGF into the lateral ventricle of adult rats, could enhance the populations of progenitors that continue to divide in the adult brain (39). Likewise, addition of EGF- and Noggin to the ciPSCLC neural induction media can produce phenotypes equivalent to primary canine neural cells including 3CB2+ radial progenitors and TUBB3+/MAP2+/NFH+/SYN+ neurons (31). For this reason, both neural induction methods were tested. Under the application of both induction methods, i.e., Noggin/EGF (Neural Induction Medium I) and EGF/bFGF (Neural Induction Medium II), NPCs maintained their expression of Nestin, PAX6, and SOX1 with no difference between the induction methods (Figure 3Aa–d). The expression of the early neuronal marker βIII Tubulin was significantly up-regulated in both groups following neural induction (Figure 3Ae,f) validating the initiation of neural differentiation. It is also worth noting that the expression of neural progenitor markers, identified by ICC, in cNPCs and hNPCs (Figure 3Ba–i) was comparable, reflecting similarities in the cellular differentiation process.

The endogenous pluripotency-related transcription factors, OCT4, SOX2, and NANOG, were analyzed via ICC in the canine and human NPCs. No expression of NANOG and OCT4 could be detected in ciPSCLC (Figure 3Ag–j) indicating successful *in vitro* differentiation of ciPSCLC into cNPCs with accompanying deactivation of the pluripotency-related pathways. To our surprise SOX2 was also not expressed in the ciPSCLC. As mentioned earlier, SOX2, a member of the SoxB1 transcription factor family, is an important transcriptional regulator of pluripotency. SOX2 is, however, also an important factor for directing the differentiation of pluripotent stem cells into neural progenitor cells and for maintaining the properties of such neural stem cells (38). In contrast our hNPCs expressed SOX2 (Figure 3Bg,h). This is a clear indication that the hiPSC can be efficiently induced into NPCs, whereas ciPSCLC may be impaired and display a lower competence for further neural differentiation.

### Differences in Terminal Neuronal Differentiation Between Canine and Human NPCs

**Figure 4:**
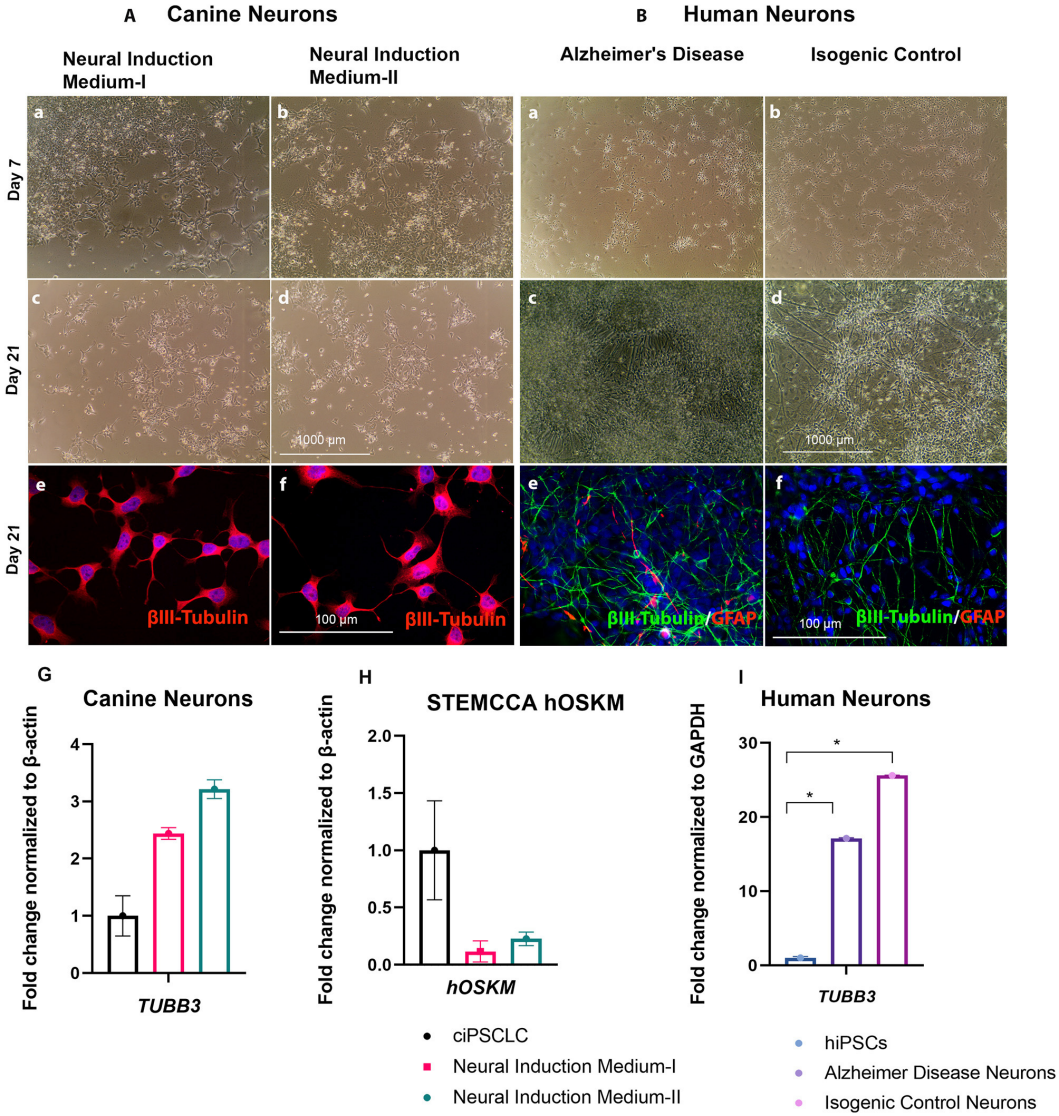
(**A**, left). Neural differentiation of cNPCs. Phase contrast pictures of live cNPCs and neurons previously exposed to Neural Induction Medium I and II at day 7 and day 21 (Scale bar = 1,000 μm). **(A)**. **(**a–d) Canine neural differentiation after neural induction in Neural Induction Medium-I (Noggin/EGF) and Neural Induction Medium-II (bFGF/EGF). Neural differentiation of NPCs after 21 days revealed immature neurons, observed with phase contract microscopy. (e,f) ICC revealing early neural marker βIII-Tubulin (red) and DNA labeled with DAPI in blue. (g) qPCR revealing *TUBB3* expression in Neural Induction Medium I and II (h) Confirmation of decreased STEMCCA (*hOSKM*) expression in cNPCs by qPCR either Neural Induction Media I or II. (Scale bar for ICC images = 100 μm, for phase contrast images = 1,000 μm). (i) qPCR revealing *TUBB3* expression. (**B**, right) (a–d) Neural differentiation of human neural progenitor cells (cNPCs) representing Alzheimer's disease and isogenic control. Neural differentiation of NPCs after 21 days revealed neurons, observed with phase contract microscope. (e,f) ICC revealing early neural marker βIII-Tubulin (red) and glial marker GFAP (green) and DNA labeled with DAPI in blue. (Scale bar for ICC images = 100 μm, for phase contrast images = 1,000 μm).

Next, we investigated if cNPCs have the ability to differentiate into mature neurons. Bright field imaging illustrates the morphological changes of both canine and human neurons over the course of the neural differentiation (Figures 4Aa–d,Ba–d). For the canine neural differentiation both protocols were implemented and compared. We then evaluated neuronal fate by performing ICC on terminal differentiated neurons at 3 weeks using varying neuronal markers. When comparing neural differentiation of cNPCs, generated with the two neural induction media, we observed no significant differences and the results are, consequently, described in common. Our differentiation experiments revealed that the majority of cells from both species were positive for βIII Tubulin, which is a well-known neuron-associated marker that is expressed in the earliest phase of neural differentiation (40). In addition to βIII Tubulin the expression of mature neuronal markers, MAP2 were analyzed. The differentiated cells lacked MAP2 expression, thereby clearly indicating that these cells were not mature neurons and are in early stage of development (Supplementary Figure 2). However, the morphology of the neurons, derived in the two species, was strikingly different. The canine neurons presented less axon fasciculation and contained shorter neurites in comparison to their human counterparts [Figures 4Ae,f (canine) and 4Be,f (human)]. Axonal fasciculation is the progression of a growing axon adhering to another, potentially forming groups of axons known as fascicles, which follow similar growth trajectories (41). The reduced axonal fasciculation in canine neurons indicates less complete neural differentiation. In addition to the ICC, we performed q-PCR to determine the expression of early neural markers. The canine neurons expressed less *TUBB3* than their human counterparts [Figures 4Ag (canine) and 4Bi (human)]. Therefore, herein we infer that incomplete silencing of exogenous vector may have influenced the differentiation efficiency in the canine model, since the expression of exogenous STEMCCA genes are still detectable (Figure 4h). Hence, overall both neuronal induction methods, applied on ciPSCLC, were suitable to generate cNPCs, but failed to generate neurons following 3 weeks of differentiation.

### Correlation Between Nuclear Size and Neural Differentiation Capacity

**Figure 5:**
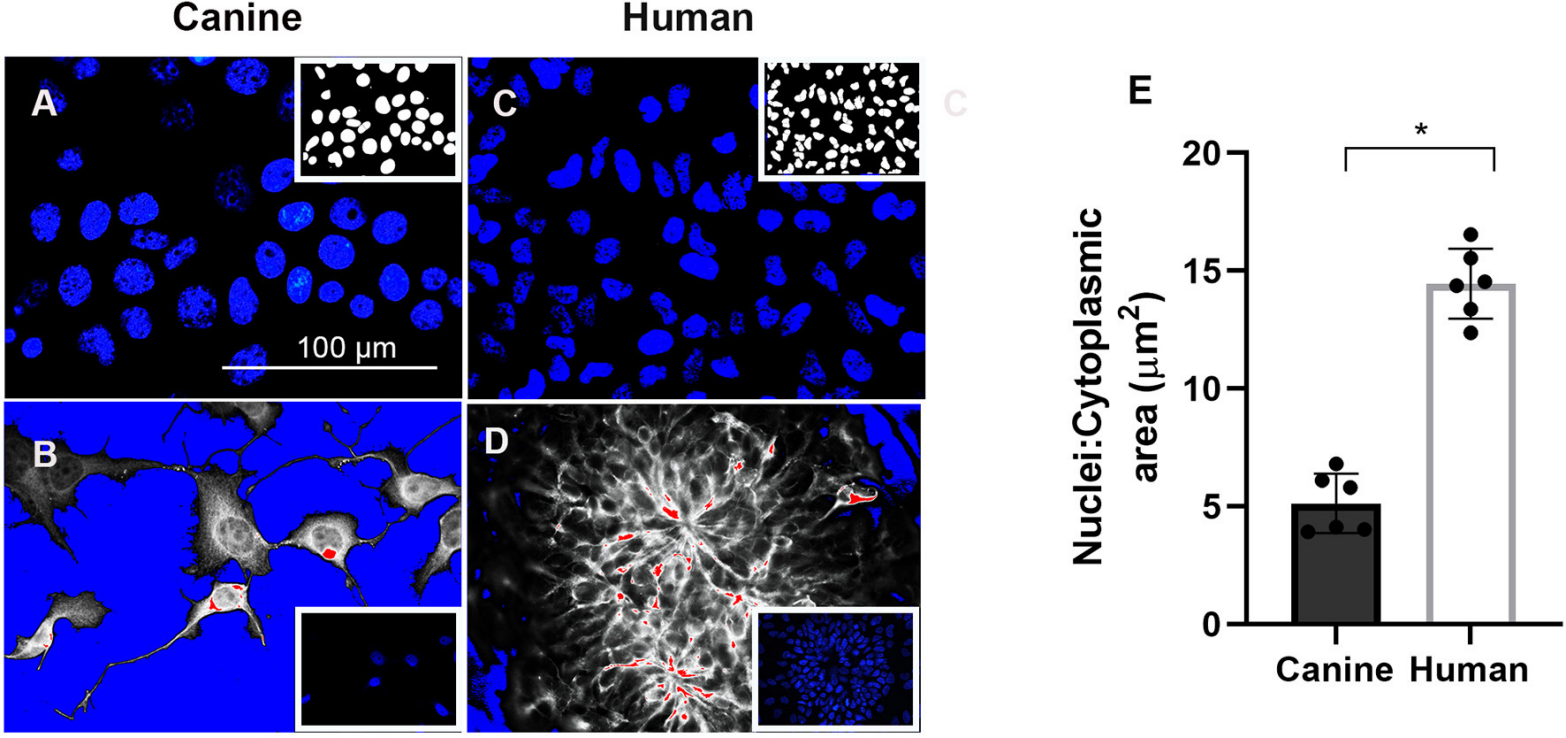
Cell nuclei were stained with (4′-6-diamidino-2-phenylindole (DAPI) in blue) after 21 days of neural differentiation. **(A)** canine nuclei. **(C)** human nuclei. Images in **(B,D)**, respectively, processed for nuclei to cytoplasmic (N/C) ratio. Scale Bar = 100 μm. **(E)** Quantification of mean N/C ratio. ^*^ < 0.05.

Considerable evidence indicates that the differentiation potential of NPCs are influenced by nuclear size and time in culture (42, 43). For example, discovery of neurodevelopmental abnormalities in lamin B1– and lamin B2–deficient mice provide new perceptions into the function of the nuclear lamina. In lamin B1 deficiency mice nuclear size, shape, and heterochromatin organization are altered (44). Therefore, we hypothesis changes in cell size that occur during development, and differentiation are accompanied by dynamic nuclear size adjustments in order to create appropriate nuclear-to-cytoplasmic volume relationships. Our canine neurons presented a larger size nucleus, as represented by Figure 5A, compared to human nucleus (Figure 5C). However, when we measured the nuclear to cytoplasmic (N/C) ratio of differentiated neurons using Image J (Figures 5B vs. 5D), we observed a significant decline of N/C ratio in canine neurons, correlating with the failed neuronal differentiation of the ciPSCLC. Furthermore, our quantification revealed that the mean size of the canine neuron is greatly reduced (N/C ratio is 5.13 μm^2^ and human neuron N/C ratio is 14.45 μm^2^) (Figure 5E). Taken together our results indicate that the nucleus: cytoplasmic ratio could influence the differentiation capacity as previously reported (45, 46).

## Discussion

Herein we report efficient induction of pluripotency in fibroblast isolated from an almost 15 years old dog presenting MCI using the OSKM lentiviral approach. Our results underline the possibility to obtain ciPSCLC from geriatric dogs, which as outlined above was so far a challenge.

Previous studies report the generation of ciPSCs from embryonic, fetal, and adult canine tissues, with the adult donor ages ranging from 7 months to 7-year-old, using integrative reprogramming systems or non-integrative sendai viruses (47). Even tough very recently one study generated a ciPSC line using episomal vectors in specific conditions (48), episomal reprogramming did not succeeded with our adult canine fibroblasts. One reason for this could be the elevated donor age, which has been related to lower reprogramming efficiency rates in some species (49–51) and has specifically reported for older dogs (52), The other reason could be divergence in episomal gene delivery. As shown recently it was possible to reprogram canine fibroblasts of undisclosed, but adult age, using a combination of different episomal plasmids (53). In our study we implemented episomal vectors carrying hOCT4, hSOX2, hKLF2, hL-MYC, hLIN28, and shTP53 genes, whilst in the other study the mouse p53 hairpin as well as additionally EBNA1 and GLIS1 were used. Especially GLIS1 has recently been shown to support pluripotency via epigenetic and metabolic remodeling (54). These recent reports are encouraging that a more complex combination of episomal plasmids could be more successful to generate ciPSC, but it is not clear from this study if it would succeed in geriatric dogs.

Our approach using the lentiviral system has resulted in ciPSCLC even though it is important to acknowledge that lentiviral integrative reprogramming methods may lead to residual expression of pluripotency factors or spontaneous reactivation of transgenes even after reported silencing. This in turn has been linked to reduced iPSC differentiation potentials, both *in vivo* and *in vitro* (55). Due to the fact that our ciPSCLCs showed similar challenges with residual expression of pluripotency factors it needs to be acknowledged that theses are only iPSC like, but not bona fide iPSC.

Previous studies have demonstrated that ciPSCs are capable of differentiation into specific cell types, such as mesenchymal and endothelial cells (56, 57). Although *in vitro* derivation of synaptic competent neurons from canine embryonic stem cells-like has long been reported (31), neuronal differentiation from ciPSCs has only recently been established, aiming at canine chronic spinal cord injury treatment (58). In the referenced study, the authors showed that injection of ciPSCs-derived NPCs in two dogs with chronic spinal cord injury did not lead to tumor formation or any other notable changes in the tissues of the spinal cord at the injection sites. Even though it appears that the risk of uncontrolled proliferation of transplanted NPCs might be low, more evidence is needed with larger numbers of animals being treated.

The other endpoint of our study was to evaluate the efficiency to obtain mature neurons. In order to investigate this we have employed two neural maturation strategies and explored similarities as well as differences between canine and human neurons derived from iPSC. The differences in efficiency of *in vitro* neural differentiation between species might indicate that the methodology still needs to be optimized for ciPSCs. This observation is reinforced by results reported in swine using a similar strategy (59). In our settings, although it was possible to obtain NPCs and neuronal-like cells from ciPSCLC, the same efficient commitment toward mature neurons as seen when differentiating hiPSCs was not acquired, which could be caused by inappropriate reactivation of pluripotency factors or integration effects. Our results revealed low level expression of OSKM in the ciPSC neurons, which could indeed stem from inproper silencing or reactivation of pluripotency factors during differentiation. Therefore, several obstacles remain. Integration-free methods are, in general, more challenging for reprogramming of fibroblasts derived from aged animals (60). At the moment non-integrative reprogramming is notoriously difficult in domestic species in contrast to mice and man. It is not clear at this point what precisely the stumbling block in the generation of bona fide pluripotent iPSC in those species are. One potential explanation could be the inefficiency of maintaining embryonic stem cells (ESCs) isolated from the inner cell mass of early blastocysts from other species besides mouse and man. Our findings indicate that culturing conditions of canine pluripotent cells needs further refinement, which poses as a big challenge for the efforts of generating and maintaining ciPSCs (61, 62).

Moreover, differentiation protocols for obtaining mature neurons need further optimization, but it is obvious that non-integrative methods could additionally improve the critical obstacle of residual pluripotency gene expression preventing proper neuronal commitment.

In conclusion, we present here ciPSCLC generated from an elderly dog with MCI, a potential useful model for *in vitro* and preclinical studies for investigating commonalities and differences related to neurodegenerative disorder in both dogs and humans. Even though the integrative approach is still challenging for subsequent differentiations, we showed that endogenous pluripotency-related genes can activated using the lentiviral OSKM reprogramming approach from canine geriatric fibroblasts. We further showed that these ciPSCLC are capable to generate putative *in vitro* neural derivatives, implementing directed differentiation protocols. Since men and dog share many similarities regarding AD pathology such a model would not only allow comparative investigations, but could also provide a very useful model to study cell replacement therapies to treat CCD and to translate those findings to humans and neurodegenerative diseases.

## Data Availability Statement

The original contributions presented in the study are included in the article/Supplementary Material, further inquiries can be directed to the corresponding author/s.

## Ethics Statement

The animal study was reviewed and approved by Ethical Committee of the Department of Veterinary Clinical Sciences, University of Copenhagen, Denmark (Permission number 2015-15-0201-00810 & 2017-7).

## Author Contributions

PH, MB, and KF performed experimental design. BT recruited and characterized the old dog where the biopsy originated from. NG performed cellular reprogramming and analyses on control cell lines. LP isolated canine cells and generated ciPSCLC. AC performed most of the experiments. JA took out the brain for evaluation and performed general necropsy of the dog. AC, BT, JA, LP, and KF interpreted the results and wrote the manuscript. FB interpreted the results, edited, and approved the paper. PH, MB, MK, and AA edited and approved the paper. All authors read and approved the final version of the paper.

## Funding

This work was supported by awards from: Independent Research Fund Denmark (FTP, grant NO. 4184-00061) and The São Paulo Research Foundation (FAPESP grant NO. 2015/26818-5 and 2017/14884-9).

## Conflict of Interest

The authors declare that the research was conducted in the absence of any commercial or financial relationships that could be construed as a potential conflict of interest.

## Publisher's Note

All claims expressed in this article are solely those of the authors and do not necessarily represent those of their affiliated organizations, or those of the publisher, the editors and the reviewers. Any product that may be evaluated in this article, or claim that may be made by its manufacturer, is not guaranteed or endorsed by the publisher.

## Supplementary Material

Supplementary Figure 1 Fibroblasts from an unaffected 4 years-old dog during episomal reprogramming. **(A,B)** The control canine fibroblast and electroporated cells after 21 days in culture. **(C,D)**. On 21th day in culture, cells showed fibroblastic morphology and formed clumps but they did not present any positive markers for OCT4, nor for the AP assay. Scale bars are 100 μm.

Supplementary Figure 2 CC revealing early neural marker MAP2 (red) and DNA labeled with DAPI in blue. The differentiated neurons failed to express mature marker MAP2, thereby clearly indicating that these cells were not mature neurons. **(A)** Canine neural differentiation after neural induction in Neural Induction Medium-I (Noggin/EGF) and **(B)** Neural Induction Medium-II (bFGF/EGF). Scale bars are 100 μm.
