# Supplementary figures and images for: Neural Derivates of Canine Induced Pluripotent Stem Cells-Like Cells From a Mild Cognitive Impairment Dog
Source: Front Vet Sci. 2021 Nov 4;8:725386. doi: 10.3389/fvets.2021.725386 (PMC8600048; doi:10.3389/fvets.2021.725386)

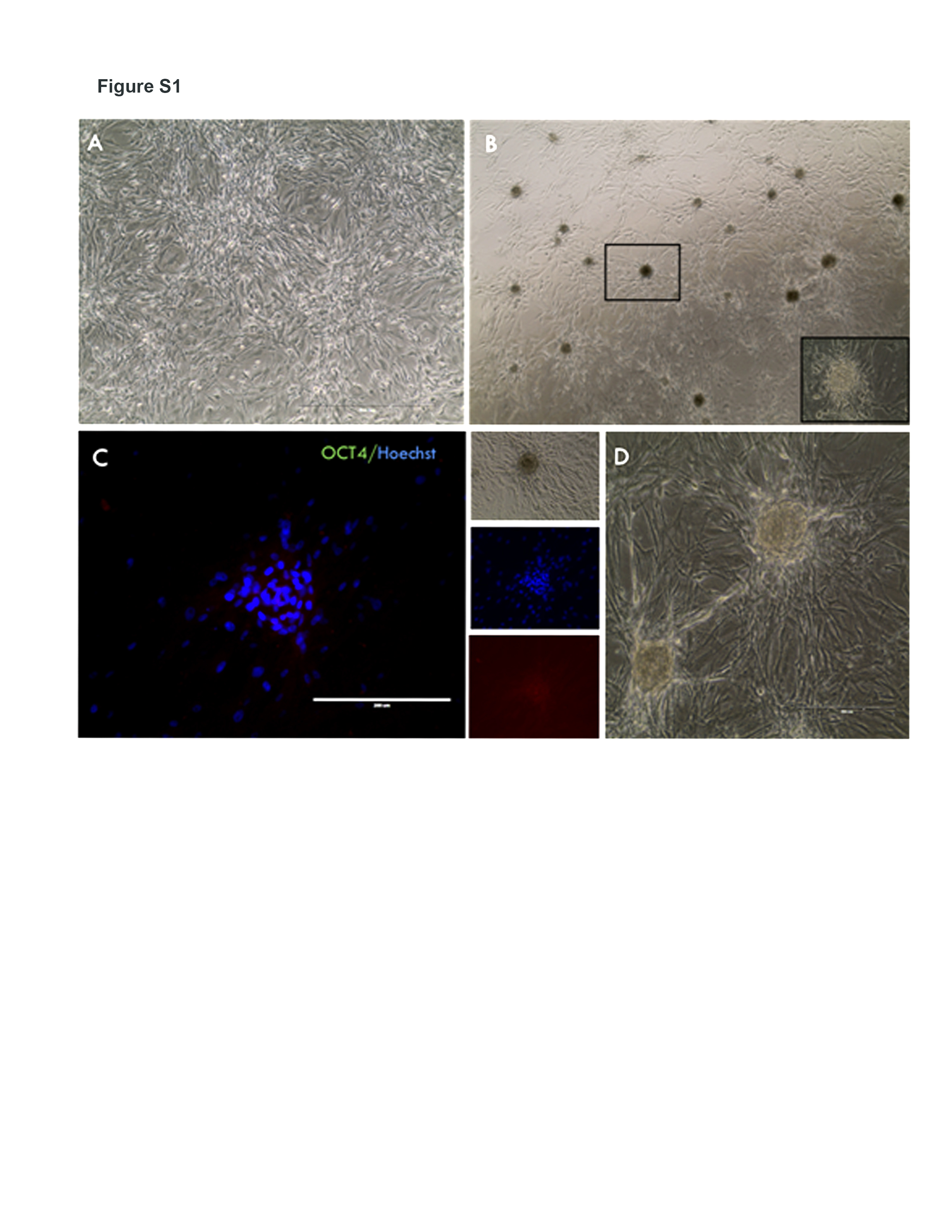

Supplement: Supplementary Figure 1 — Fibroblasts from an unaffected 4 years-old dog during episomal reprogramming. (A,B) The control canine fibroblast and electroporated cells after 21 days in culture. (C,D). On 21th day in culture, cells showed fibroblastic morphology and formed clumps but they did not present any positive markers for OCT4, nor for the AP assay. Scale bars are 100 μm. [file Image_1.jpeg]

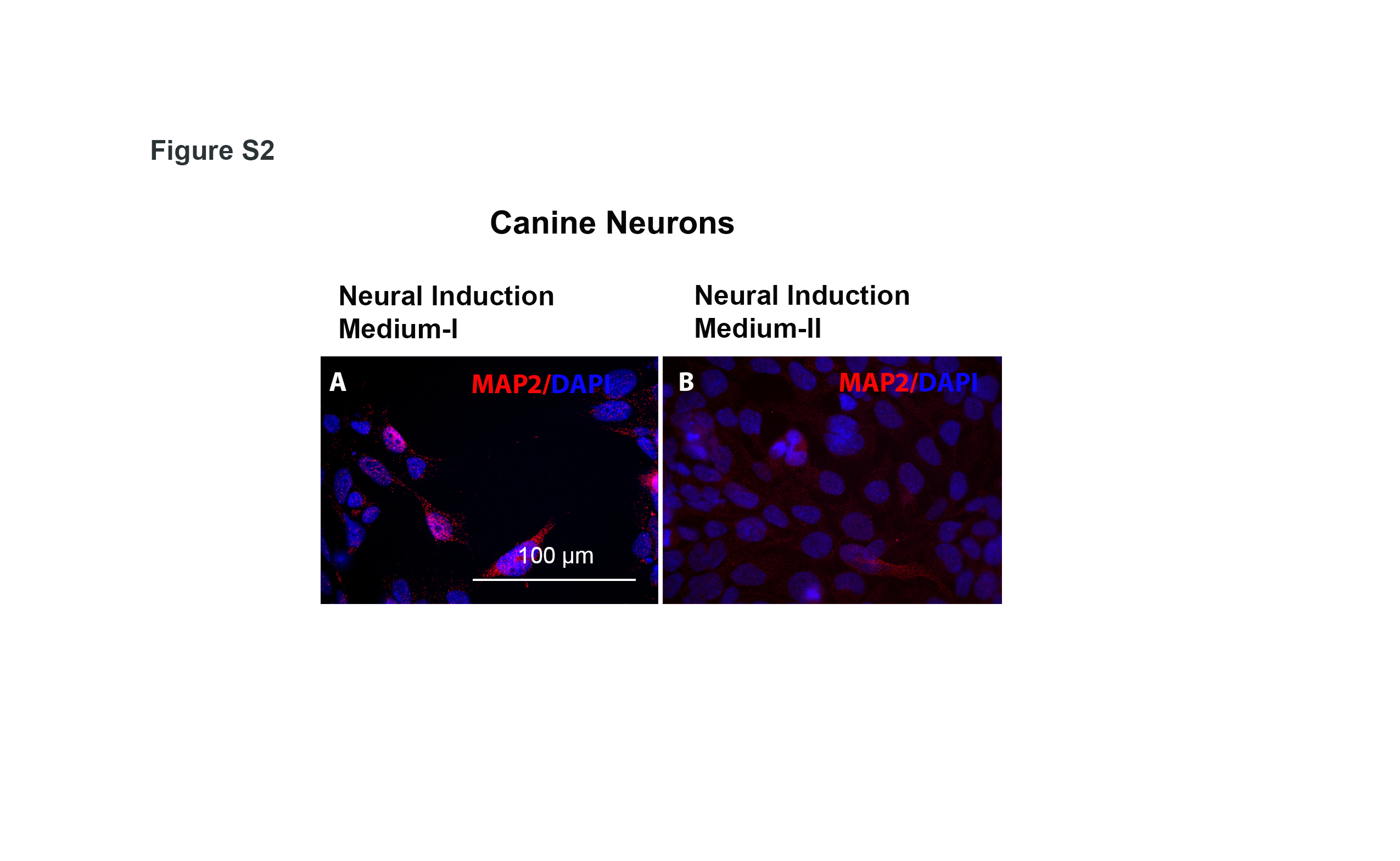

Supplement: Supplementary Figure 2 — CC revealing early neural marker MAP2 (red) and DNA labeled with DAPI in blue. The differentiated neurons failed to express mature marker MAP2, thereby clearly indicating that these cells were not mature neurons. (A) Canine neural differentiation after neural induction in Neural Induction Medium-I (Noggin/EGF) and (B) Neural Induction Medium-II (bFGF/EGF). Scale bars are 100 μm. [file Image_2.jpeg]
